# Serum-Based Oxylipins Are Associated with Outcomes in Primary Prevention Implantable Cardioverter Defibrillator Patients

**DOI:** 10.1371/journal.pone.0157035

**Published:** 2016-06-09

**Authors:** Yiyi Zhang, Eliseo Guallar, Elena Blasco-Colmenares, Amy C. Harms, Rob J. Vreeken, Thomas Hankemeier, Gordon F. Tomaselli, Alan Cheng

**Affiliations:** 1 Department of Epidemiology, Welch Center for Prevention, Epidemiology and Clinical Research, Johns Hopkins University Bloomberg School of Public Health, Baltimore, MD, United States of America; 2 Division of Cardiology, Department of Medicine, Johns Hopkins University School of Medicine, Baltimore, MD, United States of America; 3 Netherlands Metabolomics Centre, Leiden Academic Centre for Drug Research, Leiden University, Leiden, Netherlands; 4 Analytical Biosciences, Leiden Academic Centre for Drug Research, Leiden University, Leiden, Netherlands; 5 Discovery Sciences, Janssen R&D, Beerse, Belgium; University of Nebraska Medical Center, UNITED STATES

## Abstract

**Introduction:**

Individuals with systolic heart failure are at risk of ventricular arrhythmias and all-cause mortality. Little is known regarding the mechanisms underlying these events. We sought to better understand if oxylipins, a diverse class of lipid metabolites derived from the oxidation of polyunsaturated fatty acids, were associated with these outcomes in recipients of primary prevention implantable cardioverter defibrillators (ICDs).

**Methods:**

Among 479 individuals from the PROSE-ICD study, baseline serum were analyzed and quantitatively profiled for 35 known biologically relevant oxylipin metabolites. Associations with ICD shocks for ventricular arrhythmias and all-cause mortality were evaluated using Cox proportional hazards models.

**Results:**

Six oxylipins, 17,18-DiHETE (HR = 0.83, 95% CI 0.70 to 0.99 per SD change in oxylipin level), 19,20-DiHDPA (HR = 0.79, 95% CI 0.63 to 0.98), 5,6-DiHETrE (HR = 0.73, 95% CI 0.58 to 0.91), 8,9-DiHETrE (HR = 0.76, 95% CI 0.62 to 0.95), 9,10-DiHOME (HR = 0.81, 95% CI 0.65 to 1.00), and PGF_1α_ (HR = 1.33, 95% CI 1.04 to 1.71) were associated with the risk of appropriate ICD shock after multivariate adjustment for clinical factors. Additionally, 4 oxylipin-to-precursor ratios, 15S-HEPE / FA (20:5-ω3), 17,18-DiHETE / FA (20:5-ω3), 19,20-DiHDPA / FA (20:5-ω3), and 5S-HEPE / FA (20:5-ω3) were positively associated with the risk of all-cause mortality.

**Conclusion:**

In a prospective cohort of patients with primary prevention ICDs, we identified several novel oxylipin markers that were associated with appropriate shock and mortality using metabolic profiling techniques. These findings may provide new insight into the potential biologic pathways leading to adverse events in this patient population.

## Introduction

In the US, approximately 3 million people have heart failure with reduced ejection fraction and are potentially eligible for implantation of a defibrillator (ICD) for primary prevention of sudden cardiac death (SCD) [1–2]. However, among primary prevention ICD patients, only a fraction receive appropriate ICD shocks prior to death [3–4], highlighting the limited specificity of current risk stratification techniques and incomplete understanding of biological processes that trigger ventricular arrhythmias. Efforts aimed to identify serum-based protein biomarkers for prediction of appropriate ICD shocks and all-cause mortality have demonstrated only modest prognostic power [5–8]. Hence, there is substantial interest in identifying novel risk factors and prognostic markers to improve current risk prediction, disease prevention, and to guide more effective therapeutic strategies.

The metabolic milieu of cardiomyocytes, both from normal and diseased hearts, contributes to the generation of arrhythmias associated with SCD [9–11]. Recent advancement in the metabolic profiling technique allows high-throughput quantitative assessment of thousands of small-molecule metabolites found in the serum [12], and has been used to identify novel biomarkers in several disease processes including coronary heart disease and diabetes [13–14]. Several cross-sectional studies have also applied metabolic profiling in heart failure patients in whom metabolic alterations have been reported [15–16]. However, the clinical value of metabolic profiling in predicting future adverse events (e.g., ventricular arrhythmias and/or mortality) in these patients is largely unexplored.

Oxylipins, a diverse class of lipid metabolites derived from the oxidation of polyunsaturated fatty acids, are potent endogenous signaling molecules involved in the regulation of various metabolic processes such as inflammation, thrombosis, lipid management, blood pressure regulation, and hemostasis [17–19]. In an effort to explore the role of oxylipin metabolites in predicting the risk of ventricular arrhythmias and all-cause mortality, we performed metabolic profiling of baseline sera from a prospective cohort of systolic heart failure patients undergoing ICD implantation for primary prevention of SCD. The aim was to identify novel biomarkers that might serve as new predictors of adverse events and to gain additional insights into the pathophysiology of disease in this patient population.

## Materials and Methods

### Study Population, Data Collection and Follow-Up

The Prospective Observational Study of Implantable Cardioverter-Defibrillators (PROSE-ICD) is a multicenter study of systolic heart failure patients receiving a primary prevention ICD. Enrollment was conducted at four United States clinical centers from 2003 to 2013 [20]. Details of the study design have been described previously [20–21]. Among the 1,189 participants enrolled in the PROSE-ICD study, sufficient baseline blood sera for metabolic profiling of oxylipins were available in 479 subjects. This study was approved by the Johns Hopkins Investigational Review Board (IRB) and all participants provided IRB-approved written informed consent.

At enrollment, all participants underwent a comprehensive medical history, cardiovascular examination, and fasting blood collection [20]. Peripheral blood is collected and allowed to stand at room temperature for 1 hour to minimize the variance in the time from collection to processing. Blood in nonanticoagulated tubes is centrifuged at 1500 rpm for 5 minutes. The serum is then transferred into tubes containing 200 to 500 μL aliquots, frozen in liquid nitrogen and stored at −80°C.

After enrollment, patients were evaluated (either in person or by phone) every 6 months and after any patient-perceived ICD shock. For this analysis, patients were followed for events through June 1, 2015. The primary endpoint was appropriate ICD shock defined as a shock delivered for rapid ventricular tachyarrhythmias. The secondary endpoint was all-cause mortality, which was obtained by next-of-kin phone interviews and National Death Index queries. Arrhythmic events were adjudicated by two cardiac electrophysiologists blinded to patient information. Disagreements were reconciled by a third electrophysiologist.

### Metabolic Profiling

Methodologies for oxylipin analysis have been previously described [22]. For oxylipin analysis, 250 μL aliquots were taken. After thawing on ice, the samples were treated immediately with antioxidants (0.2 mg BHT/EDTA) and spiked with internal standards (ISTDs). Samples were analyzed by liquid chromatography (Agilent 1260, San Jose, CA) coupled to electrospray ionization on a triple quadrupole mass spectrometer (Agilent 6460, San Jose, CA). To detect the individual oxylipins, multiple reaction monitoring (MRM) in negative ion mode was performed with individually optimized fragmentor voltage and collision energies (Optimizer application, MassHunter, Agilent). MRM transitions were achieved by flow injection of pure standards and the optimizer application and were compared to literature when available for the certain compounds. Peak determination and peak area integration was performed with Mass Hunter Quan (Agilent, Version B.04.00) while auto-integration was manually inspected and corrected if necessary. The obtained peak areas of targets were corrected by appropriate internal standards and calculated response ratios (i.e., peak area of oxylipin target / peak area of ISTD; unit free) were used throughout the analysis.

### Statistics

Metabolites with levels below the lower limits of detection were imputed with a value of the lower limit of detection (for the given compound) divided by 2. Metabolites with a significant amount of missing values (>25%) were excluded from the analysis. Due to the skewed distribution of the oxylipins, values were log-transformed to approximate a normal distribution and subsequently standardized to have a mean of 0 and standard deviation of 1. For each oxylipin, we also calculated the oxylipin-to-precursor ratio as the ratio may provide information above and beyond their individual components (for example, the ratios may serve as a proxy for the activity of conversion enzymes). Similar to oxylipins, all oxylipin-to-precursor ratios were also log-transformed and standardized to have a mean of 0 and standard deviation of 1.

To identify associations of each oxylipin and oxylipin-precursor ratio with the study endpoints, we used the Cox proportional hazards regression to calculate the hazard ratios (HR) associated with every standard deviation (SD) change in the log-transformed oxylipin level. For all analyses, we used two models with covariates added sequentially. The first model was adjusted for age, sex, race, and enrollment center. The second model was further adjusted for ejection fraction, NYHA class, cardiomyopathy etiology, atrial fibrillation, diabetes, hypertension, and CKD. We also performed sensitivity analyses further adjusting for ECG markers (QRS, QTc) and medications (aspirin, ACE-I/ARB, beta-blocker, diuretics, and aldosterone antagonist), and found no significant differences in the results (data not shown). Since the nature of this analysis is largely exploratory, nominal p-values from the Cox regression models were reported. STATA version 12 (StataCorp LP, College Station, Texas) was used for all analyses.

## Results

The average age of participants at baseline was 60.1 ± 12.8 years. Men comprised of 72.9% of the study population and 62.6% were Caucasians (Table 1). After a median follow-up of 6.3 years, 69 participants experienced an appropriate ICD shock (incidence rate 3.1 per 100 person-years), and 161 participants died (incidence rate 5.5 per 100 person-years). The majority of participants who died did not experience an appropriate ICD shock (135 out of 161, 84%). Participants who had an appropriate shock during follow-up were less likely to be hypertensive (Table 1). Participants who died were more likely to be older, male, Caucasian, and to have NYHA Class III symptoms, ischemic cardiomyopathy, and higher burden of comorbidities including atrial fibrillation, diabetes, hypertension, and chronic kidney disease (Table 2).

**Table 1:** Baseline characteristics of participants, by appropriate ICD shock.

Characteristic	Total	No appropriate ICD shock	Appropriate ICD shock	p-value
	(n = 479)	(n = 410)	(n = 69)	
Age (year)	60.1 ± 12.8	60.2 ± 12.9	59.6 ± 12.5	0.74
Sex				0.17
Male	349 (72.9)	294 (71.7)	55 (79.7)	
Female	130 (27.1)	116 (28.3)	14 (20.3)	
Race				0.06
White	300 (62.6)	249 (60.7)	51 (73.9)	
Black	166 (34.7)	148 (36.1)	18 (26.1)	
Other	13 (2.7)	13 (3.2)	0 (0.0)	
Ejection fraction (%)	21.9 ± 7.5	21.9 ± 7.5	22.1 ± 7.3	0.86
NYHA class				0.87
Class I	70 (14.6)	59 (14.4)	11 (15.9)	
Class II	192 (40.1)	167 (40.7)	25 (36.2)	
Class III	216 (45.1)	183 (44.6)	33 (47.8)	
Class IV	1 (0.2)	1 (0.2)	0 (0.0)	
Cardiomyopathy etiology				0.27
Non-ischemic	217 (45.3)	190 (46.3)	27 (39.1)	
Ischemic	262 (54.7)	220 (53.7)	42 (60.9)	
Atrial fibrillation	119 (24.8)	104 (25.4)	15 (21.7)	0.52
Diabetes	154 (32.2)	132 (32.2)	22 (31.9)	0.96
Hypertension	289 (60.3)	258 (62.9)	31 (44.9)	0.01
Chronic kidney disease	136 (28.4)	121 (29.5)	15 (21.7)	0.25
Medications				
Aspirin	318 (66.4)	273 (66.6)	45 (65.2)	0.82
ACE-I / ARB	349 (72.9)	299 (72.9)	50 (72.5)	0.94
Beta blocker	429 (89.6)	370 (90.2)	59 (85.5)	0.23
Diuretics	333 (69.5)	290 (70.7)	43 (62.3)	0.16
Aldosterone antagonist	122 (25.5)	110 (26.8)	12 (17.4)	0.10

Values are number (%) or mean ± SD

**Table 2:** Baseline characteristics of participants, by all-cause mortality.

Characteristic	Alive	Dead	p-value
	(n = 318)	(n = 161)	
Age (year)	57.1 ± 12.2	66.1 ± 12.0	<0.001
Sex			0.001
Male	216 (67.9)	133 (82.6)	
Female	102 (32.1)	28 (17.4)	
Race			0.01
White	184 (57.9)	116 (72.0)	
Black	125 (39.3)	41 (25.5)	
Other	9 (2.8)	4 (2.5)	
Ejection fraction (%)	22.1 ± 7.7	21.6 ± 7.2	0.51
NHYA class			0.01
Class I	57 (17.9)	13 (8.1)	
Class II	132 (41.5)	60 (37.3)	
Class III	128 (40.3)	88 (54.7)	
Class IV	1 (0.3)	0 (0.0)	
Cardiomyopathy etiology			<0.001
Non-ischemic	167 (52.5)	50 (31.1)	
Ischemic	151 (47.5)	111 (68.9)	
Atrial fibrillation	59 (18.6)	60 (37.3)	<0.001
Diabetes	82 (25.8)	72 (44.7)	<0.001
Hypertension	181 (56.9)	108 (67.1)	0.03
Chronic kidney disease	63 (19.8)	73 (45.3)	<0.001
Medications			
Aspirin	205 (64.5)	113 (70.2)	0.21
ACE-I / ARB	226 (71.1)	123 (76.4)	0.22
Beta blocker	288 (90.6)	141 (87.6)	0.31
Diuretics	214 (67.3)	119 (73.9)	0.14
Aldosterone antagonist	77 (24.2)	45 (28.0)	0.38

Values are number (%) or mean ± SD

**Fig 1:**
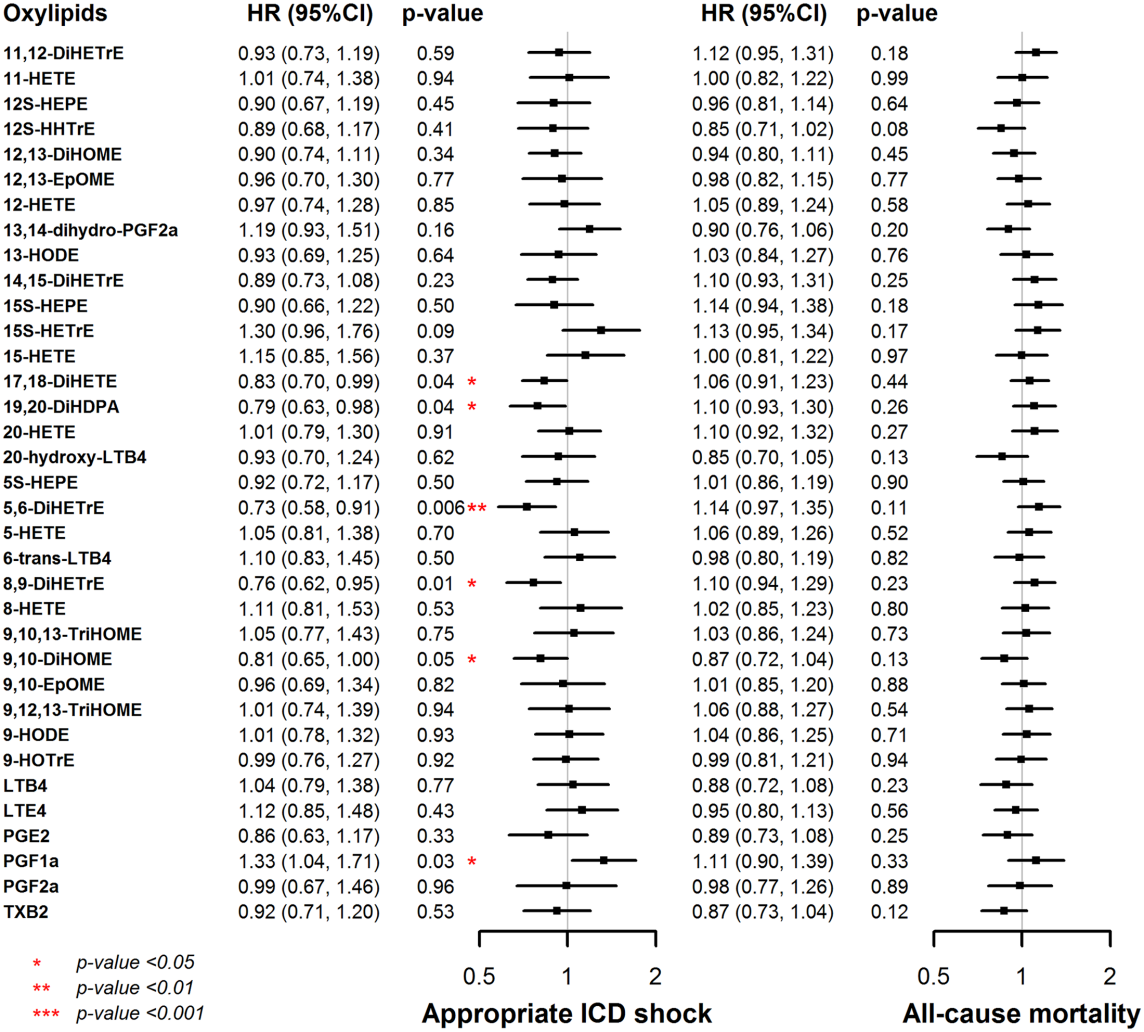
Adjusted hazard ratios (HR) and 95% confidence interval (CI) for appropriate shock and all-cause mortality associated with each oxylipin. Models were adjusted for age, sex, race, enrollment center, ejection fraction, NYHA class, cardiomyopathy etiology, atrial fibrillation, diabetes, hypertension, and chronic kidney disease.

Six oxylipins, 17,18-DiHETE (HR = 0.83, 95% CI 0.70 to 0.99 per SD change in log-transformed oxylipin level), 19,20-DiHDPA (HR = 0.79, 95% CI 0.63 to 0.98), 5,6-DiHETrE (HR = 0.73, 95% CI 0.58 to 0.91), 8,9-DiHETrE (HR = 0.76, 95% CI 0.62 to 0.95), 9,10-DiHOME (HR = 0.81, 95% CI 0.65 to 1.00), and PGF_1α_ (HR = 1.33, 95% CI 1.04 to 1.71) were associated with the risk of appropriate ICD shock after adjusting for age, sex, race, enrollment center, smoking status, body mass index, ejection fraction, NYHA class, atrial fibrillation, diabetes, hypertension, and chronic kidney disease (Fig 1). None of the oxylipins was significantly associated with the risk of all-cause mortality.

**Fig 2:**
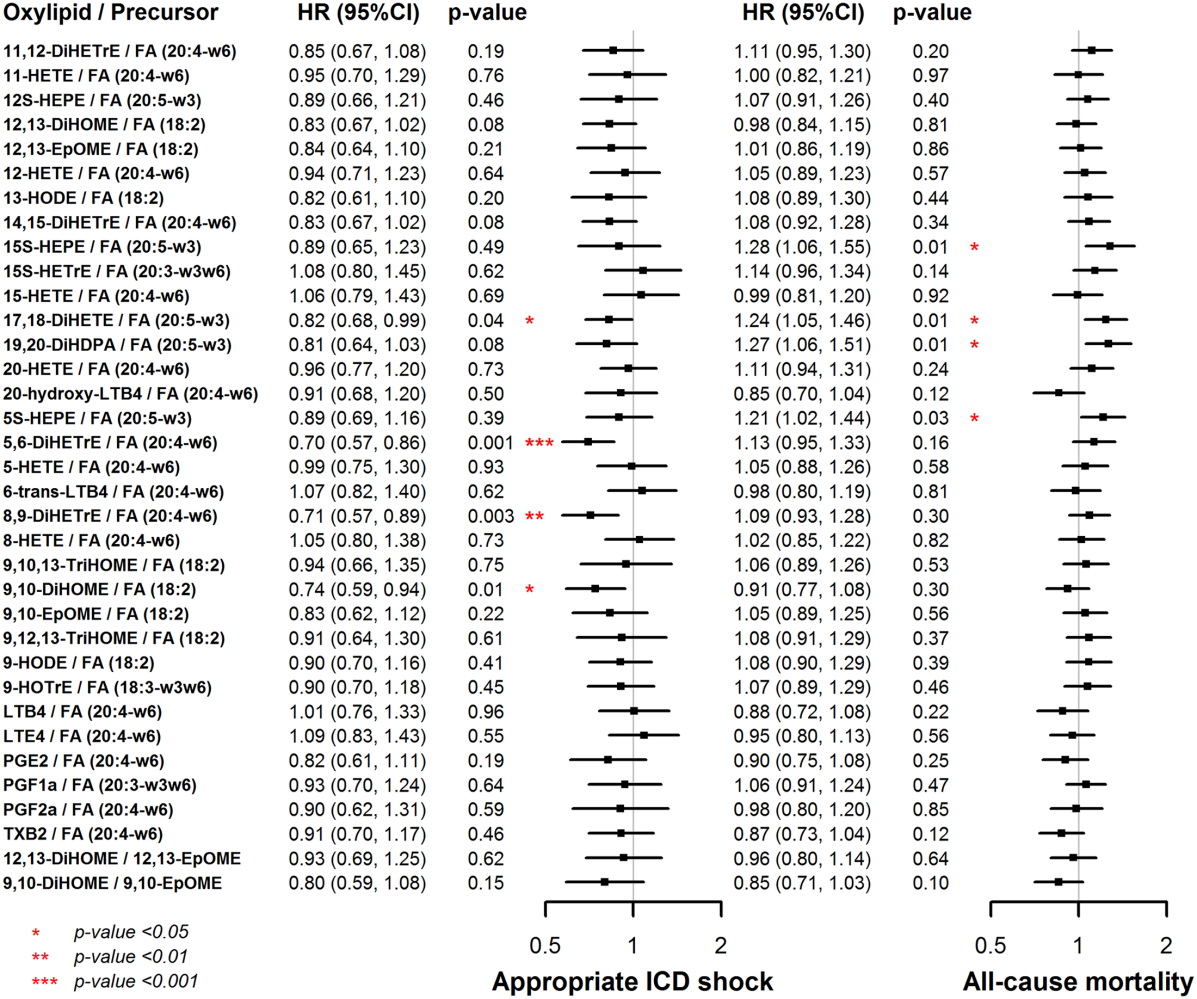
Adjusted hazard ratios (HR) and 95% confidence interval (CI) for appropriate shock and all-cause mortality associated with each oxylipin-to-precursor ratio. Models were adjusted for age, sex, race, enrollment center, ejection fraction, NYHA class, cardiomyopathy etiology, atrial fibrillation, diabetes, hypertension, and chronic kidney disease.

We found similar patterns of associations between oxylipin-precursor ratios and the risk of appropriate shocks for the oxylipins mentioned above (Fig 2). Additionally, 4 oxylipin-to-precursor ratios, 15S-HEPE / FA(20:5- ω3) (HR = 1.28, 95% CI 1.06 to 1.55), 17,18-DiHETE / FA (20:5-ω3) (HR = 1.24, 95% CI 1.05 to 1.46), 19,20-DiHDPA / FA (20:5-ω3) (HR = 1.27, 95% CI 1.06 to 1.51), and 5S-HEPE / FA(20:5- ω3) (HR = 1.21, 95% CI 1.02 to 1.44), were positively associated with the risk of all-cause mortality.

## Discussion

Using metabolic profiling, we identified 6 oxylipins (17,18-DiHETE, 19,20-DiHDPA, 5,6-DiHETrE, 8,9-DiHETrE, 9,10-DiHOME and PGF_1α_) that were associated with the risk for appropriate ICD shocks in a prospective cohort of primary prevention ICD patients, suggesting a role as novel markers of ventricular arrhythmias. Additionally, the ratios of 4 oxylipins to their precursors were positively associated with all-cause mortality. These associations remained true even after adjustment for several demographic variables.

The metabolic environment represents a collection of downstream products from various biological processes in which the cardiomyocytes are continuously exposed. Research has shown that abnormal myocardial metabolic activity and excessive oxidative stress can modulate ion channel / transporter dysfunction that predisposes to ventricular arrhythmias and SCD [11]. The largest body of evidence concerning the role of metabolites in arrhythmogenesis and SCD comes from the literature on lipid metabolism. Fatty acid levels were positively associated with SCD in a large observational study [9], and abnormalities in fatty acid metabolism were associated with sudden unexpected deaths in infants and children [23–24]. In contrast to free fatty acids and their metabolites, elevation of circulating n-3 poly unsaturated fatty acid (PUFA) levels is associated with a reduction in the risk of SCD in several observational studies [25–27].

Oxylipins are bioactive metabolites derived from the oxygenation of PUFAs such as arachidonic acid (AA), linoleic acid (LA), eicosapentaenoic acid (EPA), docosahexaenoic acid (DHA), and dihomo-γ-linolenic acid (DGLA) [22]. Different precursor PUFAs are transformed into a variety of oxylipins by three main classes of enzymes: cyclooxygenase (COX), lipoxygenase (LOX), and cytochrome P450 (CYP450) [22]. Recent advancements in mass spectrometry have allowed for quantitative evaluation of approximately 100 oxylipins down to low nanomolar concentration levels [22]. Leveraging this new lipidomic profiling technique, we identified several oxylipins that were associated with future risks of ventricular arrhythmias and mortality. These findings suggest that these oxylipins may serve as novel biomarkers or therapeutic targets for adverse events in this patient population.

Although the role of many oxylipins in cardiovascular disease is still uncertain, accumulating evidence suggests that they play an important role in the progression of cardiovascular risk factors, inflammation, and thrombosis [17, 19, 22]. Five of the 6 oxylipins identified in our study were metabolites generated through the CYP450 pathway [22]. CYP450 eicosanoids have been shown to be involved in the regulation of vascular tone, cardiac contractility, cellular proliferation, and inflammation [28]. CYP450 enzymes convert AA to a family of epoxyeicosatrienoic acids including 5(6)-epoxyeicosatrienoic acid [5(6)-EpETrE] and 8(9)-EpETrE [29]. These EpETrEs are potent modulators of vasodilatation, angiogenesis, ion conductance, and anti-inflammatory and antithrombotic processes [19, 29–30]. Once formed, they are rapidly metabolized by the soluble epoxide hydrolase (sEH) enzyme to corresponding downstream dihydroxyeicosatrienoic acids (DiHETrEs) [29]. Indeed, our study found inverse associations of 5,6-DiHETrE and 8,9-DiHETrE, stable hydrolysis products of the 5(6)-EpETrE and 8(9)-EpETrE, with the risk of appropriate ICD shock. A study in a mouse model has shown that 5,6-DiHETrE and 8,9-DiHETrE can produce dose-dependent vasodilatation by modulating the bioavailability of nitric oxide (NO) via endothelial NO synthase [31]. Furthermore, in a lipidomics study of 16 healthy male volunteers characterizing the temporal changes in peripheral blood inflammatory compounds, 5,6-DiHETrE and 8,9-DiHETrE levels were elevated after treatment with the non-steroidal anti-inflammatory drug ibuprofen, suggesting their potential role in anti-inflammatory modulation [29]. Similarly, the levels of 5,6-DiHETrE were increased after intervention with diclofenac in another study of overweight and obese men [32].

Oxylipins 9,10-DiHOME, 17,18-DiHETE and 19,20-DiHDPA are stable metabolites of LA, EPA and DHA, respectively, that are produced through the CYP450 pathways [22]. The actions of these oxylipins are still poorly understood, but some studies have shown that 17,18-EpETE, an intermediate metabolite of 17,18-DiHETE, has concentration-dependent vasodilatation effects on the pulmonary and cerebral arteries [33–34], has anti-inflammatory effects in human lung tissue [35], and exert negative chronotropic effects and protects neonatal rat cardiomyocytes against Ca^2+^ overload [36]. Additionally, 19,20-EpDPE, an intermediate metabolite of 19,20-DiHDPA, has been shown to decrease Ca^2+^ sensitivity in human pulmonary arteries [37]. Our study found inverse associations of 17,18-DiHETE and 19,20-DiHDPA with the risk of appropriate shock. Interestingly, we also observed positive associations between the ratios of 17,18-DiHETE and 19,20-DiHDPA to their precursors and all-cause mortality. These findings may suggest that a higher conversion rate of these oxylipins might be associated with worse survival, but further experimental and clinical studies are needed to better understand the underlying mechanisms.

PGF_1α_ is a biosynthesis product of DGLA via the COX pathway [22]. In our study, it was the only oxylipin that was positively associated with the risk of appropriate ICD shocks. Prior studies have reported that PGF_1α_ could modulate the contraction and relaxation of vascular smooth muscle from the arterial strips [38] and increase coronary blood flow and myocardial contractile force [39]. In an anesthetized dog model, injection of PGF_1α_ intravertebrally caused an increase of blood pressure and tachycardia, whereas intravenous or intracarotid infusion of the same dose of PGF_1α_ had no effect. These findings suggest that PGF_1α_ has little direct effect on heart rate but may affect heart rate indirectly through a reflex stimulation or inhibition on the sympathetic and vagal nerves [40–41]. Beyond these observations, the exact mechanism underlying the association between PGF_1α_ and appropriate shock is unknown.

Additionally, we found positive associations between the ratios of 5S-HEPE and 15S-HEPE to their respective precursors and the risk of mortality. Both 5S-HEPE and 15S-HEPE were metabolites of EPA via the LOX pathway [22]. These LOX metabolites may have anti-inflammatory effects and play an important role in the resolution phase of inflammation [42]. In a lipidomics study of 5 patients undergoing cardiac surgery, levels of 5-HEPE were increased 24 hours after the surgery compared to before surgery [22]. It has also been shown that individuals with hyperlipidemia had higher levels of 5-HEPE [18]. Similarly, animal study has demonstrated the potential anti-inflammatory property of 15-HEPE in inflammatory skin disorders [42].

Several limitations of our study need to be considered. Since our study was by nature observational, we could only identify associations but not establish causal links between oxylipins and outcomes. Although our study included a large cohort of systolic hear failure patients with primary prevention ICDs, it may be still underpowered to detect associations with outcomes as relatively few patients experienced these events. Given the nature of our cohort, findings from this analysis may not be applicable to patients at high risk for sudden death but with preserved left ventricular function.

## Conclusions

In a prospective cohort of patients with primary prevention ICDs, we identified several novel oxylipin markers that were associated with appropriate shock and all-cause mortality using metabolic profiling technique. Additional studies are required to confirm these findings in other patient populations and to better understand the exact mechanisms underlying the associations between oxylipins and SCD.

## Abbreviations

AA: arachidonic acid
CKD: chronic kidney disease
COX: cyclooxygenase
CRT: cardiac resynchronization therapy
CYP450: cytochrome P450
DGLA: dihomo-γ-linolenic acid
DHA: docosahexaenoic acid
ECG: electrocardiogram
eGFR: estimated glomerular filtration rate
EPA: eicosapentaenoic acid
ICD: implantable cardioverter-defibrillator
ISTD: internal standard
LA: linoleic acid
LOX: lipoxygenase
MRM: multiple reaction monitoring
NYHA: New York Heart Association
PUFA: poly unsaturated fatty acid
PROSE-ICD: Prospective Observational Study of Implantable Cardioverter-Defibrillators
SCD: Sudden cardiac death
